# *Trypanosoma rangeli* turns infected mice more attractive to triatomine bugs

**DOI:** 10.1098/rspb.2026.2486

**Published:** 2026-11-11

**Authors:** Luan F. Santos, Marcelo G. Lorenzo, Renan P. de Souza, Alessandra A. Guarneri

**Affiliations:** ^1^ Grupo de Comportamento de Vetores e Interação com Patógenos, Instituto René Rachou, Fiocruz, Belo Horizonte, MG, Brazil; ^2^ Instituto de Investigaciones en Biodiversidad y Biotecnología (INBIOTEC-CONICET), Mar del Plata, Buenos Aires, Argentina; ^3^ Instituto René Rachou, Fiocruz, Belo Horizonte, MG, Brazil; ^4^ Departamento de Genética, Ecologia e Evolução, Universidade Federal de Minas Gerais, Belo Horizonte, MG, Brazil

**Keywords:** *Trypanosoma cruzi*, behavioural manipulation, host–pathogen interaction

## Abstract

Manipulation of host behaviour is a common strategy used by parasites to increase transmission. Both *Trypanosoma cruzi* and *Trypanosoma rangeli* are known to alter the behaviour of their vectors, but little has been reported concerning their effects on the vertebrate hosts. The present study investigated whether infection with these trypanosomes could induce enhanced attractiveness of mammalian hosts to triatomine bugs. Assays showed that mice infected with *T. rangeli* attracted significantly more *Rhodnius prolixus* nymphs than uninfected controls (*p* < 0.0001), whereas no significant effect was induced by *T. cruzi*. Furthermore, despite higher approach rates, nymphs approaching *T. rangeli*-infected mice were preyed upon less often than those approaching control mice (*p* = 0.005). Notably, 46.5% of the nymphs that fed on *T. rangeli*-infected mice acquired the parasite, confirming its efficient transmission. This combination of increased attraction and reduced predation creates an optimal scenario for transmission by *T. rangeli*. Together, these results indicate that *T. rangeli* infection alters host–vector interactions in a manner potentially beneficial to parasite transmission, establishing a new model for investigating trypanosomatid effects on vertebrate hosts.

## 1. Introduction

The evolutionary dynamics of host–parasite interactions are characterized by strong selective pressures. While parasites are selected to successfully infect hosts and exploit their resources for survival and reproduction, hosts are selected to avoid infections or mitigate their fitness costs. Host manipulation is a common parasite strategy to enhance parasite fitness, usually by increasing transmission to the subsequent host [1–3]. Such parasite-induced phenotypic alterations have been documented across a wide range of host and parasite taxa [3–8], including humans [9]. Nevertheless, not all infection-associated changes necessarily represent cases of adaptive manipulation. Demonstrating manipulation requires showing that the parasite induces a change in host phenotype that increases parasite fitness and is not merely a pathological by-product of infection [2,10]. Consequently, studies investigating putative manipulation often begin by identifying parasite-associated alterations that could enhance transmission and then evaluate whether these alterations are consistent with the parasite life cycle and transmission strategy.

One mechanism frequently associated with enhanced transmission is the alteration of host traits that increase proximity between intermediate and definitive hosts [11–14]. Several parasites are known to modify vertebrate host phenotypes in ways that increase their attractiveness to blood-feeding arthropods. For example, infections by *Plasmodium* and *Leishmania* can increase the attractiveness of vertebrate hosts, including humans, to mosquitoes and sand flies, thereby enhancing transmission opportunities [15–18].

*Trypanosoma cruzi* is a protozoan parasite, which circulates between mammals and blood-feeding insects of the subfamily Triatominae (Hemiptera, Reduviidae). When infecting humans, it causes Chagas disease, a neglected tropical disease affecting seven million people worldwide, mostly in Latin America [19]. The related parasite *Trypanosoma rangeli* shares mammalian and triatomine hosts with *T. cruzi* but is considered non-pathogenic to humans. Nevertheless, infection by *T. rangeli* induces antibodies that cross-react with *T. cruzi*, potentially interfering with diagnostic procedures used in public health programs [20,21].

Unlike *T. cruzi*, *T. rangeli* exhibits a highly specialized transmission cycle. Although it infects a broad diversity of mammalian hosts, including humans, rodents, bats, marsupials and primates, successful transmission depends primarily on species of the genus *Rhodnius*, in which the parasite invades the hemocoel and salivary glands, and differentiates into infective metacyclic forms [22–25]. Furthermore, only a fraction of infected vectors develop salivary gland infections capable of transmitting the parasite [26–29]. This dependence on a restricted group of vectors may impose constraints on transmission efficiency and potentially favour the evolution of traits that increase contact rates between infected hosts and susceptible vectors.

As nocturnal insects, triatomines remain hidden in shelters during daylight hours to find protection from predators and to interact with conspecifics [30]. Host-seeking motivation reaches its maximum during the first hours of the scotophase, under the control of an endogenous circadian clock [31]. To locate hosts, triatomines integrate multiple chemical and physical cues, including humidity, heat, odours and exhaled CO_2_ [30,32]. Lorenzo & Lazzari [33] investigated the shelter-use dynamics of fasting *Triatoma infestans* nymphs and reported increased exit rates at the onset of the scotophase, with returns to the shelter occurring more intensely a few hours before dawn. Ferreira *et al.* [34] showed that only a small proportion of *Rhodnius prolixus* nymphs left their shelters in the absence of host cues, even after 60 days of fasting. In the presence of host-derived odours, this proportion rose to approximately 25%, suggesting that robust host-related signals are required to induce *R. prolixus* nymphs to come out of their shelters [34].

*Rhodnius prolixus* is considered the most relevant vector of Chagas disease in the Andean region, especially in Colombia and Venezuela [35,36]. The species exhibits a strong capacity for domiciliation, enabling efficient transmission of the parasite in both wild and domestic environments [35,37,38]. In addition to transmitting *T. cruzi*, *R. prolixus* is highly susceptible to *T. rangeli* infection and supports the complete development of the parasite, including salivary gland colonization and the production of infective metacyclic forms.

Both *T. rangeli* and *T. cruzi* can alter the physiology and behaviour of *R. prolixus*, even promoting vector pathogenicity [39]. In the case of *T. cruzi* infection, activity alterations lead to increased bug predation rates by mice, a phenomenon that may facilitate transmission through the oral route [40,41]. Regarding *T. rangeli*, infected *R. prolixus* exhibits prolonged feeding contact with vertebrate hosts, a behaviour partly attributed to the parasite usage of salivary proteins during gland colonization [42,43]. In addition, *T. rangeli* infection promotes increased locomotor activity, reduced negative phototaxis and a higher propensity to leave shelters to seek hosts [44,45]. These changes increase the frequency of host–vector encounters and may consequently enhance parasite transmission. Whether these parasites also induce phenotypic changes in their vertebrate hosts that could affect interactions with triatomine vectors remains unknown.

In the present study, we investigated whether mice infected with *T. cruzi* or *T. rangeli* alter the interaction between mammalian hosts and *R. prolixus*. Specifically, we evaluated whether infected mice were approached more frequently by triatomine nymphs and whether infection affected host defensive behaviour towards the insects. By examining how infection influences host–vector interactions, we aimed to assess whether these changes could potentially increase transmission opportunities for these parasites.

## 2. Material and methods

### (a) Organisms

The *R. prolixus* specimens used in this study were obtained from a colony derived from insects collected in Honduras in the 1990s and maintained by the Grupo de Comportamento de Vetores e Interação com Patógenos of the Instituto René Rachou. The insects were kept under semi-controlled conditions of temperature (26 ± 1°C) and relative humidity (57 ± 10%) and exposed to the natural light cycle. The colony was maintained with citrated sheep or rabbit blood obtained from the Instituto de Ciência e Tecnologia em Biomodelos, FIOCRUZ, Rio de Janeiro, Brazil and offered at 37°C by means of an artificial feeder.

Sixty-day-old female Swiss Webster mice, weighing approximately 40 g, were obtained from the animal facility. When necessary, the animals were anesthetized intraperitoneally with a mixture of Ketamine 150 mg kg^–1^ (Cristália–Brazil) and Xylazine 10 mg kg^–1^ (Bayer–Brazil), in a final volume of approximately 100 µl per animal, depending on mouse weight. The Dm28c *T. cruzi* strain, isolated from a naturally infected *Didelphis marsupialis* [46], and the CHOACHI *T. rangeli* strain, originally isolated from a naturally infected *R. prolixus* [47], were used. Parasites were cultured at 27°C by performing two weekly passages in liver infusion tryptose medium supplemented with 15% fetal bovine serum and antibiotics (100 units ml^–1^ of penicillin and 100 µg ml^–1^ of streptomycin). To maintain infectivity, parasites were subjected to triatomine and mouse infection every three months [48].

Mouse infection with *T. cruzi* was promoted by an intraperitoneal inoculation of 200 µl of *R. prolixus* urine containing metacyclic trypomastigotes. Urine was obtained as described by Guarneri [48]. Briefly, fifth-instar nymphs previously infected with *T. cruzi* were blood-fed and immediately placed individually into sterile 1.5 ml microcentrifuge tubes with perforated caps and maintained at 27°C. Urine was collected within 1–2 hours post-feeding. This procedure ensures an inoculum containing approximately 1 × 10^5^ par ml^–1^. For the control group, mice with similar characteristics of age and weight were inoculated with urine of uninfected insects, ensuring that all animals underwent similar experimental procedures. Animals were used on the 9-day post-inoculation [49]. Infection with *T. rangeli* was promoted using fifth-instar *R. prolixus* nymphs that had been previously infected and exhibited metacyclic trypomastigotes in their salivary glands. An infected nymph releases approximately 50 000 metacyclic *T. rangeli* during a blood meal, and a single nymph bite is sufficient to establish infection in mice [50]. To maximize infection success, anesthetized mice were exposed to the bites of approximately 10 to 15 infected nymphs for 10 minutes. For the control group, mice were exposed to bites of uninfected nymphs. Rodents were subsequently used 14 days post-infection [51].

### (b) Evaluation of host attractiveness in mice infected with *Trypanosoma cruzi* or *Trypanosoma rangeli*

To assess whether infection with *T. cruzi* or *T. rangeli* alters the attractiveness of mice to *R. prolixus* nymphs, two sets of assays were conducted (supplementary material, figure S1A). The first tested the attractiveness of mice infected with *T. cruzi*, while the second evaluated the attractiveness of *T. rangeli*-infected mice, maintaining consistent experimental conditions in both cases. Groups of 50 fifth-instar nymphs were used in the experiment. The insects had been fed during the fourth instar and, after molting to the fifth instar, were maintained without feeding for approximately 30 days. For each trial, insects were released at the end of the photophase into a glass arena (50 × 50 × 20 cm^3^) presenting Kraft paper as substrate. An artificial shelter made of a 20 × 10 cm^2^ piece of corrugated cardboard folded in half was placed at the centre of the arena. After folding, the structure formed a 10 × 10 cm refuge with two lateral entrances approximately 0.5 cm high [33]. The insects remained in the arena for 72 hours to acclimate to the environment and to ensure they all accessed the shelter.

After this interval, two cylindrical plastic containers (10 cm high × 8 cm in diameter), sealed with a metal mesh, were placed near one corner of the arena, 8 cm apart from each other, one containing an infected mouse and the other a non-infected control. The containers prevented physical contact between the insects and the mice but allowed the dissemination of chemical (odours) and physical (vibration, water vapor and heat) stimuli, revealing host presence. Three strips of double-sided tape were applied horizontally around each container, while another was fixed around its base to capture approaching and climbing insects, following the principle of the Noireau trap [52].

Mice were placed in the arena 3 hours before the onset of the scotophase on the last day of acclimation, remained there throughout the entire scotophase, and were removed 1 hour after the start of the following photophase (with *ad libitum* access to food and water during the entire assay). After this 16-hour interval, the number of nymphs stuck on the tapes of each container was recorded. The position of infected and uninfected mice was alternated between trials to avoid position bias. Insects and mice were used only once.

The experiments were recorded using IP cameras (model 6310; Yoosee, Shenzhen, China) that are infrared-light-sensitive and provide active infrared illumination (built-in IR LEDs), with a wavelength not perceived by triatomines [53]. Two cameras were used to allow a complete visualization of bugs and mice throughout the experiment. One camera was positioned above the containers and the other in front of them. In this way, the number of insects approaching each container, as well as mouse activity, could be recorded. An insect was considered to be near the host when it remained within 2 cm of the container holding the mouse for at least 15 s. To analyse this parameter, the number of insects in this position was counted at the end of each 30-minute interval over the 16-hour recording. The experiments were conducted in a room with controlled temperature and lighting conditions (12:12 L/D and 24 ± 1°C). Fifteen and 21 replicate assays were carried out with mice infected with *T. cruzi* or *T. rangeli*, respectively. At the end of each trial, blood samples from infected mice were examined for the presence of parasites. When parasites were not detected by fresh blood examination, xenodiagnosis was performed because *T. rangeli* parasitemia is typically low. In these cases, uninfected nymphs were allowed to feed on the animals, and their intestinal contents were examined 30 days later [50].

### (c) Evaluation of nymph predation rates by mice infected with *Trypanosoma rangeli*

The evaluation of predation rates was carried out following the methodology described in the previous experiment, with modifications (supplementary material, figure S1B). Two glass arenas were used, and a group of 50 fifth-instar *R. prolixus* nymphs, starved for 30 days, was released into each arena. The insects remained there for a 72-hour acclimation interval. Three hours before the onset of the scotophase on the last day of acclimation, a caged mouse infected with *T. rangeli* was placed in one of the arenas, while a caged healthy mouse was presented as a control in the other arena. The metal wire cages had 1 cm spacing between bars, allowing direct interaction between the insects and the mice, i.e. nymphs could blood-feed and mice could predate bugs. The trial was concluded 1 hour after the beginning of the next photophase (total duration: 16 hours). The control and infected mice were alternated between the two arenas across trials.

The percentage of insects predated by mice was calculated at the end of each experiment by counting the number of nymphs remaining in each arena. All nymphs were weighed together prior to the assay, and an average individual weight was calculated. After the experiment, fed nymphs were individually weighed and the amount of blood ingested was estimated by subtracting the initial average weight. Nymphs fed on infected mice were separated to assess transmission rates. Positivity in the insects was evaluated 30 days after the end of the assays (except for the first repetition, where positivity was evaluated 21 days after completion) through optical microscopy analysis of hemolymph and intestinal content samples. Hemolymph was obtained by excising one of the tarsi, and the intestinal tract was dissected and macerated in 20 µl of PBS (0.15 M NaCl in 0.01 M sodium phosphate, pH 7.4). Nymph approach rates were assessed as previously described.

### (d) Evaluation of mouse activity during assays

Mouse activity was quantified to evaluate whether infection altered host behaviour during the assays (*n* = 10). For this purpose, video recordings obtained during these assays were analysed. The containers were covered with a perforated acrylic lid that allowed mice to be observed through its transparency. The total time spent resting and moving was recorded. Movement periods were further categorized as feeding behaviour or other activities, including scattering bedding or feces outside the container and exploring the perforations of the lid with the snout.

### (e) Statistical analysis

For all experiments, each assay was treated as an independent experimental unit because a new group of insects and a new pair of mice were used in every assay, with no reuse of animals across experiments. Data on insect captures, considering the sum of captures of all assays, were analysed using the chi-square test. The Wilcoxon test (for two paired samples), which allows assessing whether the difference between groups is consistent across experiments while accounting for experimental variability, was used considering individual results from each trial.

A binomial mixed-effects model with a logit link was fitted to evaluate the relative preference for infected versus control mice in the approach behaviour assays. At each observation interval, the number of insects found near the infected mouse was modeled relative to the total number of insects found near either mouse. Thus, the model estimated the probability that an insect approaching one of the two hosts was found near the infected rather than the control mouse. Observation intervals in which no insects approached either mouse were excluded because preference could not be defined for those intervals. Time was included as a continuous fixed effect and centred at its mean. This parametrization allowed the model intercept to represent the estimated preference at the average observation time, while the coefficient associated with time quantified any change in preference over the observation period. Trial was included as a random intercept to account for the repeated-measures structure within each experiment. Model adequacy was assessed using simulated residuals, including tests of uniformity, dispersion and outliers, implemented with the DHARMa package.

A generalized linear mixed model (GLMM) with a Poisson distribution and a logarithmic link function was used to analyse insect approach data in the experiment assessing predation rates. Unlike the host attractiveness experiment, insects in this set-up could be preyed upon by the mice, so the number of insects available for approach behaviour decreased progressively over the course of each trial. To correct the number of events (approaches) considering the number of insects available, the logarithm of the mean insect availability during each trial was used, calculated as: mean availability = (quantity available at the beginning + quantity available at the end)/2, where the quantity available at the end = 50 – quantity predated. This correction prevents the progressive reduction in insect availability from being confounded with genuine changes in approach behaviour. Random effects included the trial (to account for variation among experimental rounds) and time (to control dependence among measurements collected at different time points within the same trial).

The percentage of nymphs that fed on mice, as well as the estimated blood intake, in the predation rate experiment was analysed using Student's *t*-test. Mouse activity was analysed using a generalized estimating equations (GEE) model with a Gaussian distribution and an identity link function [54]. As the same individuals were measured repeatedly over time, the GEE approach was used to explicitly account for within-individual dependence, ensuring that the reported standard errors and *p*-values are statistically valid. The model included infection status (infected versus control) and behavioural category (resting/active time and eating/performing other activities) as fixed effects. An exchangeable correlation structure was specified to account for the dependence of repeated measures taken from the same individual (ID) over time. Interaction terms were initially tested but removed from the final model owing to non-significance (*p* > 0.05).

All analyses were performed using R software (version 4.5.1, R Foundation for Statistical Computing, Vienna, Austria). Statistical differences were considered significant when *p* < 0.05. Full model outputs used in the analysis, including fixed-effect estimates, standard errors and random-effect variance components, are provided in the supplementary material (santos_statistical_models).

## 3. Results

### (a) Approach to mice infected with *Trypanosoma cruzi*

All insects were found inside shelters after acclimation, and few of them left the shelter in the presence of light, even after mouse introduction. The few individuals that abandoned the shelter restricted their movements to the corners of the arena and failed to interact with the host. After lights off, many nymphs left the shelters, foraged and approached mice. Furthermore, individuals repeatedly attempted to climb the containers, concentrating in the area of the arena where mice were located.

A total of 750 nymphs were evaluated across 15 replicates; of these, 474 (63.2%) remained inside the shelter at the end of the assays, while 276 (36.8%) were found outside the shelter. The number of individuals recorded outside the shelter varied across the assays, with some more than 70% of nymphs leaving the shelter and others with less than 15% (supplementary material, figure S2). However, in 73.3% of the assays, the proportion of nymphs that left the shelter during mouse presentation did not reach 45% of the total.

Excluding bugs captured on tape, 81 insects that came out of the shelter during the assay did not return with the onset of the photophase. Eighty-six nymphs were captured near containers with *T. cruzi*-infected mice, while 109 nymphs were stuck near containers with control mice. When assays were considered altogether, no significant difference was detected in the number of nymphs captured near the containers (figure 1A; $\chi^{2}$ = 2.7128, d.f. = 1, *p* = 0.0995; *n* = 15). Similarly, when considering the individual values from each trial, no significant difference was observed (figure 1B; Wilcoxon's test; *p* = 0.0721; *n* = 15). During the analysis of the recordings, we observed that not all insects that approached the containers were captured by the adhesive tapes. The model-estimated probability of approaching the *T. cruzi*-infected mouse remained slightly below 50% throughout the observation period (figure 1C). Overall, there was evidence of a modest preference for the control mouse (*β* = −0.103, SE = 0.052, *z* = −2.001, *p* = 0.045), corresponding to an estimated probability of approximately 47.4% of approaching the *T. cruzi*-infected mouse (and 52.6% of approaching the control mouse) among insects that approached either animal. There was no evidence that this preference changed over time (*β* = 0.0004, SE = 0.0134, *z* = 0.027, *p* = 0.978).

**Figure 1.**
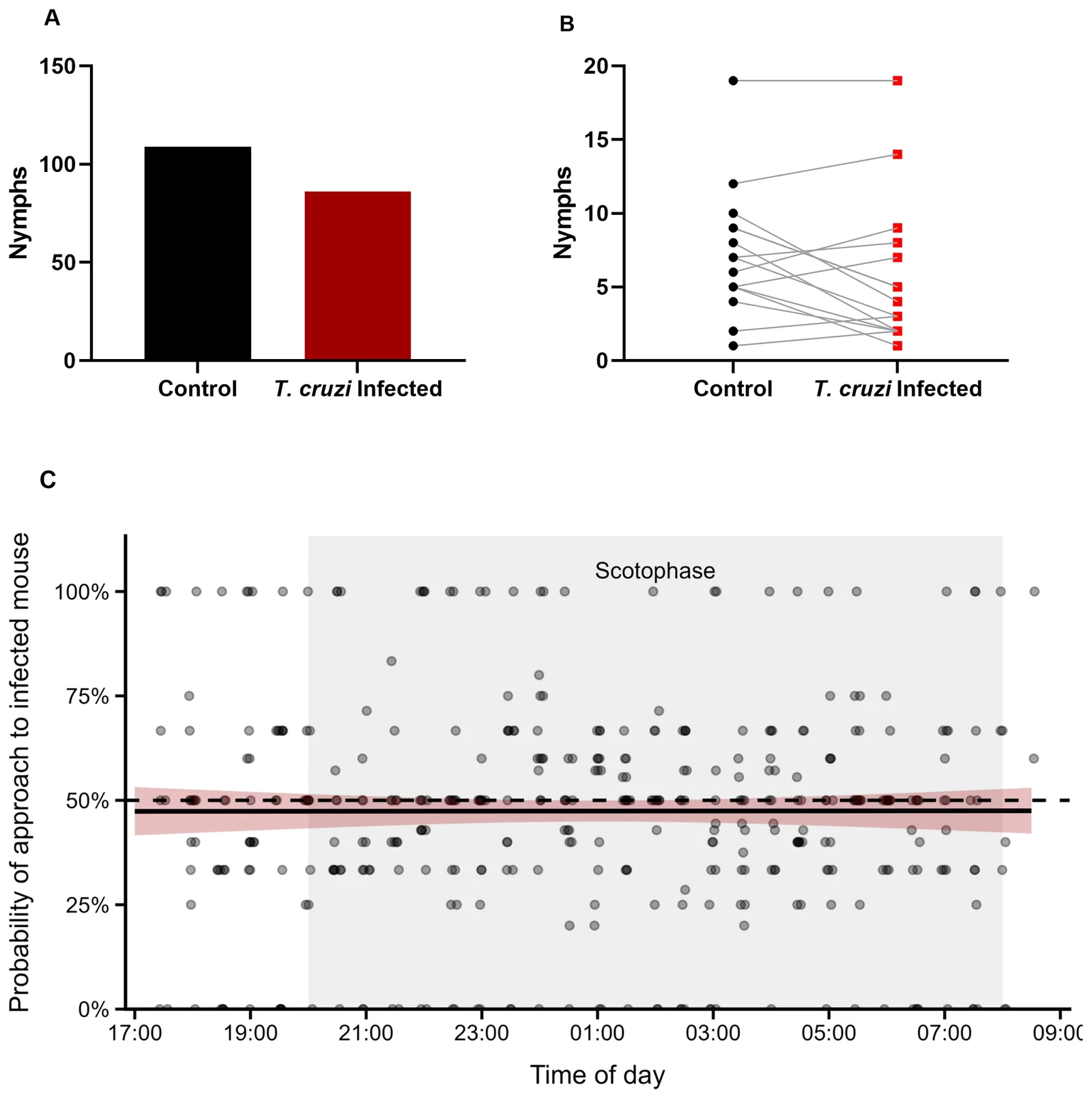
Approach to *T. cruzi*-infected mice by *R. prolixus* nymphs. (A) Total number of *R. prolixus* nymphs captured by the adhesive tapes placed on containers holding mice infected or not infected with *T. cruzi* ($\chi^{2}$ = 2.7128, d.f. = 1, *p* = 0.0995; *n* = 15). (B) Individual data points of *R. prolixus* nymphs captured by adhesive tapes placed on containers holding mice infected or not infected with *T. cruzi* in each trial; lines connect paired data points from the same assay (Wilcoxon's test, *p* = 0.0721). (C) *Rhodnius prolixus* nymphs found near the container holding the *T. cruzi*-infected mouse relative to those approaching either mouse throughout host presentation. Points represent the observed proportion at each observation interval. The solid line represents the probability estimated by the binomial mixed-effects model, with the red shaded area indicating the 95% confidence interval. The dashed line at 50% indicates no preference between *T. cruzi*-infected and control mice. Overall, nymphs showed a modest but statistically significant preference for the control mouse (*p* = 0.045).

### (b) Approach to mice infected with *Trypanosoma rangeli*

All insects in this experiment entered the shelter during acclimation. Furthermore, after mouse presentation, bugs showed a behavioural pattern similar to that observed in the *T. cruzi* experiment, with a higher rate of shelter exit following lights-off. At the end of the assays, 317 (30.2%) out of 1050 insects evaluated were found outside the shelter. Seventy-seven of them (7.3% of the total and 24.3% of the insects outside the shelter) were distant from the mice, i.e. not captured on adhesive tapes. The number of insects found outside the shelter, regardless of whether they were captured on the adhesive tapes or free in the arena, also varied considerably between replicates (supplementary material, figure S3). Regarding nymphs captured on adhesive tape, 154 were near containers holding infected mice and 86 near containers with control mice. The number of insects captured near *T. rangeli*-infected mice was significantly higher (figure 2A; $\chi^{2}$ = 19 267; d.f. = 1; *p* < 0.0001). When the assays were evaluated separately, thus allowing us to assess whether the difference between groups was consistent across trials, the number of nymphs near *T. rangeli*-infected mice was also significantly higher (figure 2B; Wilcoxon's test, *p* = 0.002). The model-estimated probability of approaching the *T. rangeli*-infected mouse remained above 50% throughout the observation period (figure 2C). Overall, there was evidence of a significant preference for the *T. rangeli*-infected mouse (*β* = 0.520, SE = 0.124, *z* = 4.203, *p* < 0.001), corresponding to an estimated probability of approximately 62.7% of approaching the infected mouse among insects that approached either animal. There was no evidence that this preference changed over time (*β* = 0.0175, SE = 0.0298, *z* = 0.587, *p* = 0.557).

**Figure 2.**
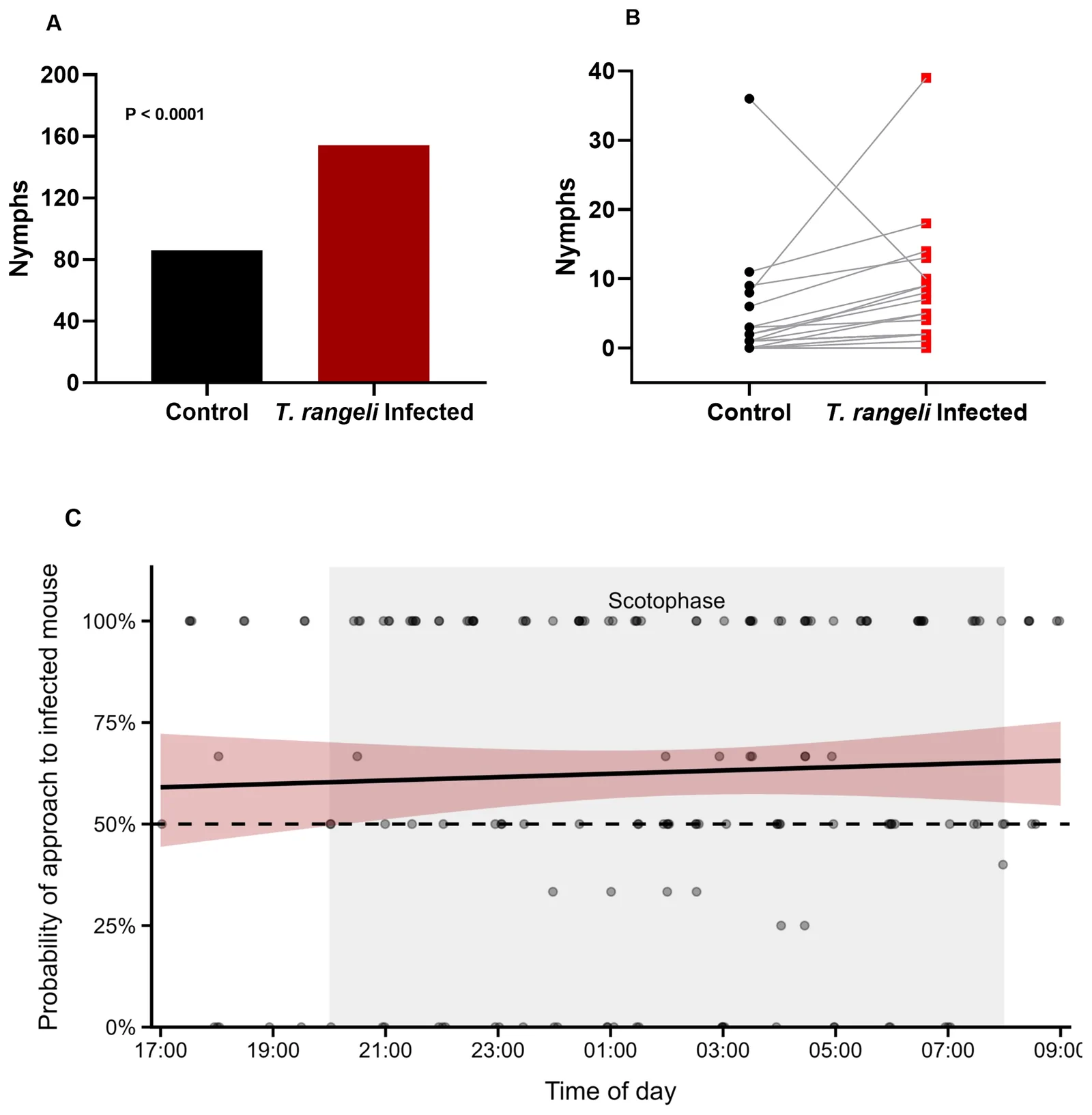
Approach to *T. rangeli*-infected mice by *R. prolixus* nymphs. (A) Total number of *R. prolixus* nymphs captured by the adhesive tapes placed on containers holding mice infected or not infected with *T. rangeli* ($\chi^{2}$ = 19.27; *p* < 0.0001; *n* = 21). (B) Individual data points of *R. prolixus* nymphs captured by adhesive tapes placed on containers holding mice infected or not infected with *T. rangeli* in each trial; lines connect paired data points from the same assay (Wilcoxon's test, *p* = 0.002). (C) *Rhodnius prolixus* nymphs found near the container holding the *T. rangeli*-infected mouse relative to those approaching either mouse throughout host presentation. Points represent the observed proportion at each observation interval. The solid line represents the probability estimated by the binomial mixed-effects model, with the red shaded area indicating the 95% confidence interval. The dashed line at 50% indicates no preference between *T. rangeli*-infected and control mice. Overall, nymphs showed a significant preference for the *T. rangeli*-infected mouse (*p* < 0.001).

### (c) Evaluation of nymph predation rates by mice infected with *Trypanosoma rangeli*

Shortly after mice were placed in each arena (one with an infected mouse and the other with a control mouse), before the onset of the scotophase, some insects were already seen leaving the shelter. At the end of the assays, at the beginning of the photophase, a few nymphs remained in the arena near the mice at 08:00 hours, but none were found close to the animals at 09:00 hours (figure 3A). Even in the model that accounts for the gradual decrease in insect numbers seen over the course of the experiment owing to predation by the mice, proportionally more nymphs approached *T. rangeli*-infected animals (figure 3A; GLMM Poisson; RR = 0.87, 95% CI = 0.77–0.99; *p* = 0.034). From the 450 nymphs tested for each condition, 103 nymphs (22.89%) were preyed upon by control mice and 66 nymphs (14.67%) by infected mice, resulting in 169 nymphs preyed upon (18.78% of the total 900) (figure 3B). When predation rates were compared, control mice preyed on a significantly higher number of nymphs (figure 3B; GLMM Poisson, *p* = 0.005).

**Figure 3.**
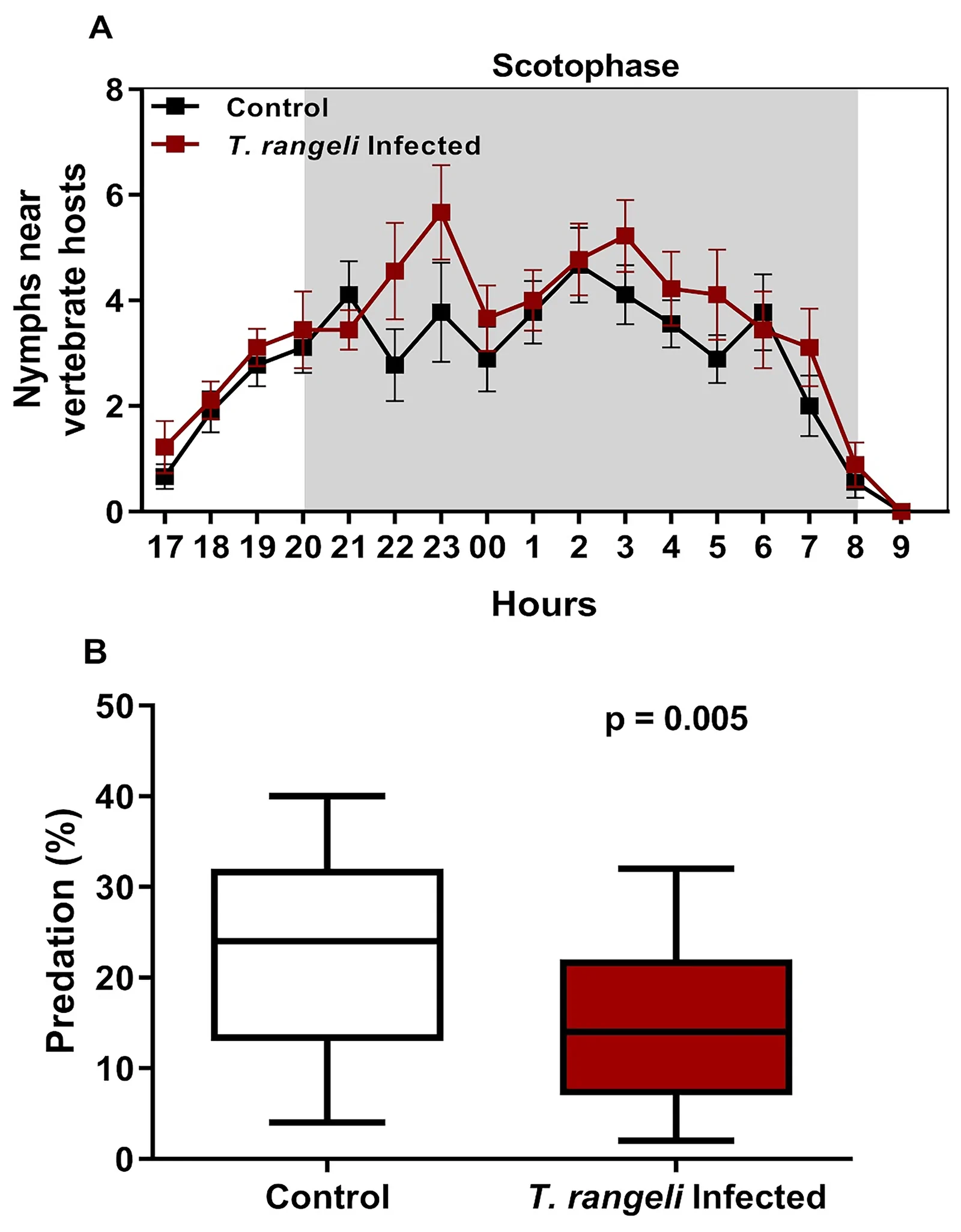
*Trypanosoma rangeli* infection decreases mouse predation on *R. prolixus* nymphs. (A) Number of nymphs found near cages holding infected and control mice in predation assays. Data are shown as mean ± standard error of the mean (s.e.m.) of nine replicates. Results were analysed using a GLMM with Poisson distribution and mean insect availability as offset (GLMM Poisson; *p* = 0.034). (B) Percentage of *R. prolixus* nymphs predated in nine independent assays. Data are shown as the median (horizontal line), interquartile range (25–75%) and min–max (Poisson's test; *p* = 0.005).

Among the surviving insects, no significant differences were observed in the proportion of nymphs that successfully fed on control (8.1 ± 1.3) or infected mice (11.9 ± 1.9) (Student's *t*-test, *p* = 0.12). Similarly, the estimated blood intake did not differ significantly between insects feeding on control (287.22 ± 42.92 mg) and infected mice (303.07 ± 38.57 mg) (Student's *t*-test, *p* = 0.18). All mice, except one, were able to transmit *T. rangeli* to 46.5% of the nymphs that fed on them (table 1). It is noteworthy that in the case of the mouse that did not transmit the parasite, the nymphs were examined 21 days after the end of the assay, whereas the other examinations were conducted within 30 days after assay completion. At the examination, none of the insects presented parasites in the hemolymph.

**Table 1.** Number of *R. prolixus* nymphs that fed on mice infected with *T. rangeli* and were found positive for parasites in their intestinal content.

vertebrate host	positive insects (analysed insects)	percentage (%)
1	0 (4)	0
2	3 (9)	33.3
3	3 (6)	50.0
4	1 (4)	25.0
5	3 (4)	75.0
6	4 (6)	66.7
7	3 (5)	60.0
8	1 (2)	50.0
9	2 (3)	66.6
**mean ± s.e.m.**	**2.22 ± 0.43**	**47.4 ± 8.0**

### (d) Mouse activity during assays with *Trypanosoma rangeli*-infected mice

To investigate whether the increased approach of triatomines to *T. rangeli*-infected mice could be associated with infection-induced changes in host behaviour, we analysed video recordings obtained during these assays. Specifically, we compared the activity patterns of infected and uninfected mice to determine whether differences in movement intensity could potentially influence bug orientation. None of the activities evaluated differed significantly between *T. rangeli*-infected and control mice (supplementary material, figure S4; GEE; not significant).

## 4. Discussion

The present study demonstrates that infection of mice with *T. rangeli* alters the behavioural interactions shown by *R. prolixus* nymphs under laboratory conditions. In approach assays, nymphs interacted with infected mice more frequently than uninfected controls, whereas no comparable effect was observed with mice infected by *T. cruzi*. Furthermore, insects interacting with *T. rangeli*-infected mice experienced lower predation rates while still acquiring the parasite efficiently. Together, these findings indicate that *T. rangeli* infection modifies traits of a model host that influence vector behaviour, increasing opportunities for parasite transmission.

The mechanisms underlying the increased approach of bugs to *T. rangeli*-infected mice remain unknown. Host-seeking triatomines use multiple sensory modalities to locate vertebrate hosts, integrating thermal, chemical and humidity-related cues [30,32]. Traditionally considered a transient parasite in mammals, *T. rangeli* has been shown to persist for extended periods in experimentally infected mice, accumulating particularly in lymphoid organs, such as the spleen and lymph nodes [55,56]. These findings suggest that the vertebrate host phase may play a more important role in parasite biology than previously recognized. Interestingly, the body temperature of infected mice was reported to increase slightly during the course of infection [56]. This observation is particularly relevant because heat constitutes one of the main short-range cues used by triatomines during host location. Furthermore, the perception of an abrupt increase in heat triggers the proboscis extension reflex in triatomines, a prerequisite for successful blood feeding [57]. Consequently, infection-induced alterations in host thermal profiles may contribute to the increased approach rates observed in the present study.

In addition to thermal cues, triatomines can also locate hosts using airstreams carrying the volatile compounds they emit. Carbon dioxide, ammonia, lactic acid, octenol, aldehydes, ketones and carboxylic acids are among the cues promoting host-seeking behaviour in blood-feeding insects. These cues are excreted products of metabolism and aid triatomines, mosquitoes, sand flies and other blood-feeding insects to find their hosts [32,58–62]. Increased attractiveness of mammalian hosts to their insect vectors has been reported in *Leishmania* and *Plasmodium* infections [16–18]. For example, in bioassays with female *Lutzomyia longipalpis,* the odour of dogs infected with *Leishmania infantum* was significantly more attractive to blood-seeking female sand flies [63]. In another study, involving dogs infected with *L. infantum*, the animals were used in laboratory host-choice assays and were found to be more attractive to female *Phlebotomus perniciosus* (vectors of visceral leishmaniasis) than uninfected dogs [18]. This result was replicated in field trials conducted in an endemic area with abundant *P. perniciosus*, where a significantly higher attraction of the insects to infected dogs was observed [18]. Similar phenomena have been described for malaria parasites. In an experiment involving 50 Kenyan children naturally infected with *Plasmodium falciparum*, *Anopheles gambiae* mosquitoes were more attracted to infected children than to those without the infection [64].

By using host-choice arenas, our approach allowed insects to respond to the full suite of host-derived cues promoting host location. However, the specific cues responsible for the increased approach were not investigated. Future studies combining behavioural assays with measurements of volatile compounds, carbon dioxide emission, body temperature and other host-derived cues will be important for identifying the mechanisms involved. Although the specific cues altered by *T. rangeli* remain unknown, our findings place this parasite within a broader group of vector-borne pathogens capable of modifying host attractiveness.

Based on video recordings obtained during the approach assays, we also evaluated whether infected mice displayed altered activity patterns that could promote differential insect attraction. However, no significant differences were detected in the time mice spent resting, moving, feeding or performing other activities. These results suggest that major changes in locomotor activity are unlikely to explain the increased attraction of bugs to infected animals. Parasite-induced alterations in vertebrate behaviour have been reported in several systems. For example, mice infected with *Plasmodium chabaudi* exhibit reduced defensive behaviour and become less likely to kill mosquitoes during feeding [65]. Likewise, infection with *Trypanosoma brucei* alters circadian rhythms and activity patterns in mice [66]. Therefore, although our analyses did not reveal significant differences in general activity, the possibility that *T. rangeli* modifies specific behavioural traits relevant to host–vector interactions remains open, especially since specific behavioural analysis methods were not applied. Interestingly, the lower insect predation rates shown by infected mice suggest that these animals may respond differently to approaching insects, a possibility that deserves further investigation.

The reduction in predation suffered by nymphs approaching infected mice is potentially relevant from an epidemiological perspective, since parasite transmission depends not only on encounters between hosts and vectors but also on vector survival during these interactions. In the present study, insects approaching *T. rangeli*-infected mice were less likely to be killed while retaining the opportunity to acquire the parasite through blood feeding. Nevertheless, caution is required when interpreting this finding as evidence of parasite-induced host manipulation. The reduced predation of nymphs by infected mice may not reflect an adaptive parasite strategy, but rather a non-adaptive, pathological side effect of infection, since sick or debilitated animals could exhibit weaker defensive responses towards approaching nymphs independently of any manipulative mechanism. Regardless of the underlying mechanism, the combined effects of increased bug attraction and reduced predation could increase opportunities for parasite transmission, and our results should be interpreted as evidence that infection alters host–vector interactions in a manner potentially beneficial to transmission rather than as definitive proof of parasite-induced manipulation.

The absence of an evident effect of *T. cruzi* infection on host attractiveness is also noteworthy. While we recognize that this does not necessarily imply that *T. cruzi* cannot make mammal hosts more attractive, this result suggests that the phenomenon observed here is not a general consequence of trypanosome infection. One possible explanation may lie in the distinct evolutionary histories and transmission strategies of these parasites. *Trypanosoma cruzi* is highly successful in circulating among a broad diversity of mammalian hosts and triatomine vectors across a wide range of ecological and geographical conditions [67]. Although many parasites are eliminated in the anterior midgut of triatomines, surviving forms differentiate and colonize the posterior gut, where metacyclogenesis occurs efficiently [68–70]. In addition, oral transmission is thought to represent an important route in sylvatic environments, contributing substantially to parasite circulation in natural cycles [41]. Together, these characteristics suggest that *T. cruzi* already possesses highly efficient transmission pathways and, therefore, may experience weaker selective pressures favouring mechanisms that increase host attractiveness.

By contrast, *T. rangeli* faces a more restrictive transmission scenario. Under such conditions, any mechanism capable of increasing encounters between infected mammals and competent vectors could potentially provide substantial transmission benefits. As previously shown, *T. rangeli*-infected *R. prolixus* nymphs display increased locomotor activity and are more likely to leave shelters to seek a blood meal [44,45,71], which increases transmission odds since the bite itself is sufficient to achieve host infection. Here, we demonstrate that the infected mammalian host became more attractive to nymphs. This feature made a higher proportion of nymphs come out of their shelters and approach the infected host (figures 2 and 3A), a characteristic that is not easily promoted in *R. prolixus* [34,72]. Taken together, these reports strongly suggest that the behaviour and physiology of triatomine vectors and mammalian hosts are altered in parallel by *T. rangeli* infection, supporting the hypothesis that *T. rangeli* may benefit from enhancing host–vector contact rates.

Finally, some limitations of our study should be acknowledged. First, the experiments were performed under controlled laboratory conditions, which may not fully reproduce the ecological complexity of natural transmission systems. The experiments were performed using mice of the genus *Mus*, which are not recognized as natural hosts of *T. rangeli*. Although natural infections by *T. rangeli* have been reported in several sylvatic rodent species, including *Trinomys dimidiatus*, *Coendou prehensilis*, *Phyllomys dasythrix (*reported as *Echimys dasythrix* in [73]*)*, *Rhipidomys* spp., *Sciurus stramineus* and *Aegialomys xanthaeolus*, no confirmed natural infection has been documented in *Mus* populations [25,73–75]. Nevertheless, the murine model provides a valuable and highly controlled system for investigating host–parasite interactions and has been instrumental in advancing our understanding of *T. rangeli* biology. In addition, the use of only females in the experiments limits the generalization of the results owing to potential differences in sex-related outcomes and behavioural responses in the context of infection. Therefore, while caution should be exercised when extrapolating these findings directly to natural transmission cycles, the model remains appropriate for identifying infection-associated phenotypic changes and generating hypotheses regarding transmission ecology. Future studies employing mammalian species naturally involved in the transmission cycle of *T. rangeli* will be important to evaluate the ecological relevance of the patterns observed here.

Despite these limitations, the present study reveals a previously unrecognized aspect of the interaction between trypanosomatids, mammalian hosts and triatomine vectors. To our knowledge, this is the first demonstration that infection of a mammalian host is associated with increased attraction of triatomine bugs. While similar phenomena have been reported for mosquito- and sand fly-borne parasites, such as *Plasmodium* and *Leishmania*, evidence of infection-associated changes influencing attractiveness to triatomines has been lacking. Given the central importance of host-seeking behaviour for parasite transmission, identifying infection-associated alterations that modify host–vector encounters represents an important contribution to our understanding of trypanosomatid ecology.

## Supplementary Material



## Data Availability

All data are in the supplementary material [76].
