# Supplementary material for: Trypanosoma rangeli turns infected mice more attractive to triatomine bugs
Source: Proc Biol Sci. 2026 Nov 11;293(2082):20262486. doi: 10.1098/rspb.2026.2486 (PMC13751123; doi:10.1098/rspb.2026.2486)
Supplement: Supplementary file 1 [file rspb.2026.2486.zip › rspb.2026.2486/rspb20262486_si_001.pdf]

Authors: Luan Felipe Santos; Marcelo Gustavo Lorenzo; Renan Pedra de Souza; Alessandra Aparecida Guarneri[1]

Title: *Trypanosoma rangeli* turns infected mice more attractive to triatomine bugs

Journal name: Proceedings B

Article DOI: 10.1098/rspb.2026.2486.

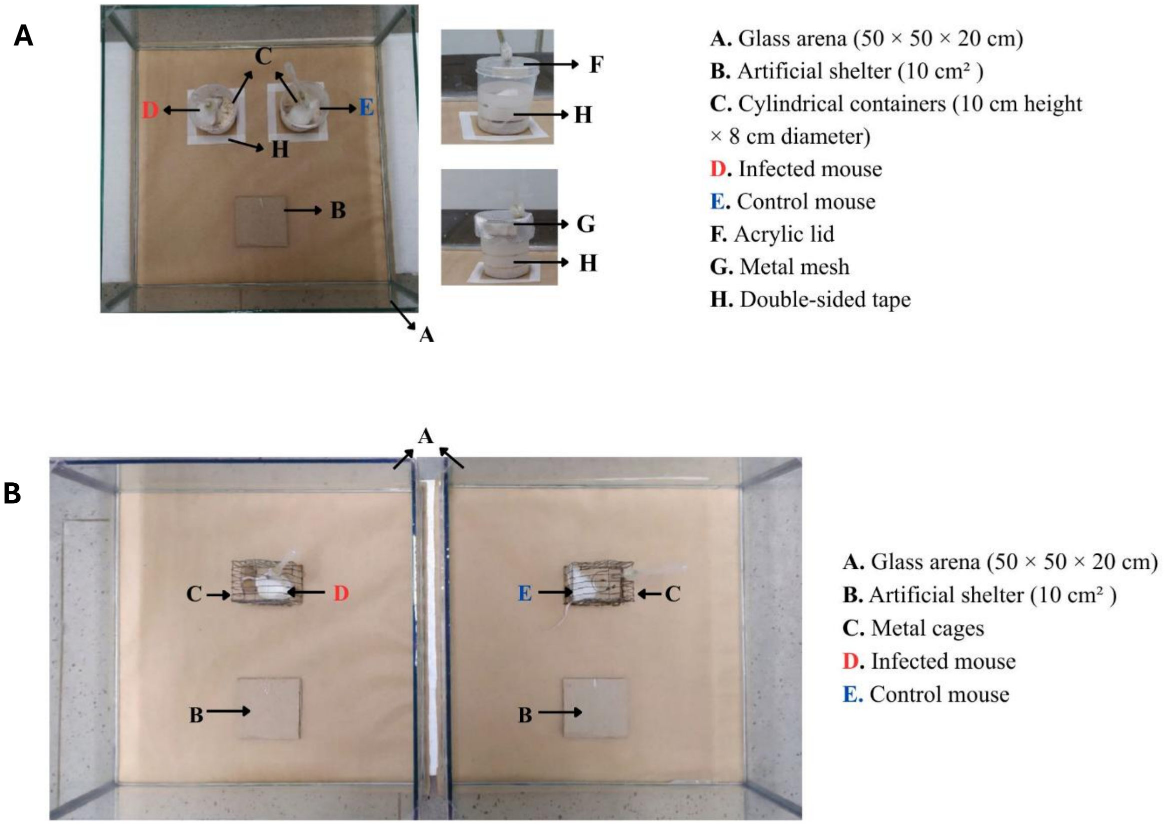

Supplementary Figure 1. Experimental arenas used to evaluate host attractiveness and predation. **A**. Glass arena (50 x 50 x 20 cm) used in the attractiveness assay, showing the artificial shelter (10 cm²), the cylindrical containers (10 cm height x 8 cm diameter) used to individually house the infected and control mice, closed with an acrylic lid and a metal mesh and fixed to the arena floor with double sided tape. **B**. Glass arenas (50 x 50 x 20 cm) used in the predation assay, each containing an artificial shelter and a metal cage housing the infected or the control mouse.

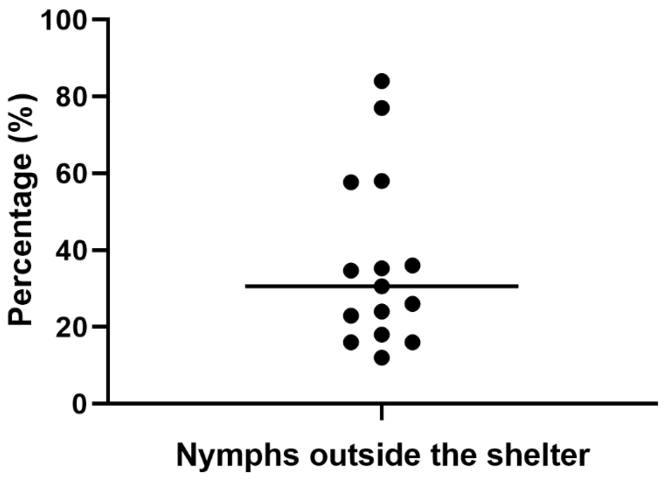

Supplementary Figure 2. Percentage of *Rhodnius prolixus* nymphs found outside the shelter at the time of host removal. Each dot represents the percentage of insects outside the shelter in one of the repetitions. The horizontal line indicates the median (n = 15).

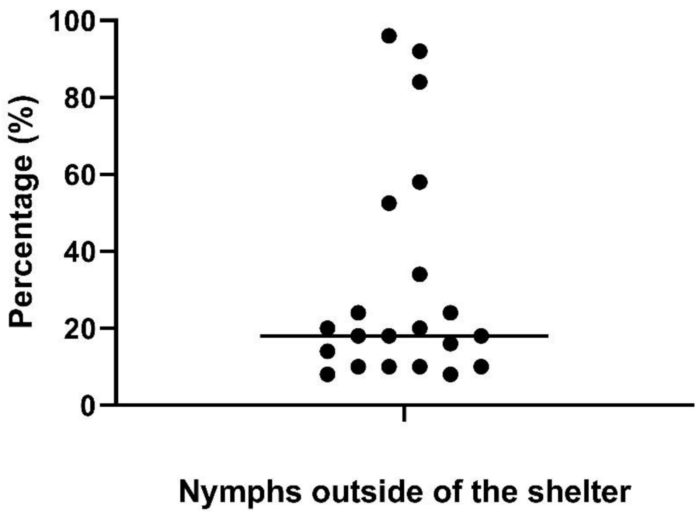

Supplementary Figure 3. Percentage of *Rhodnius prolixus* nymphs found outside the shelter at the time of host removal. Each point represents the percentage of insects outside the shelter in one of the repetitions. The horizontal line indicates the median (n = 21).

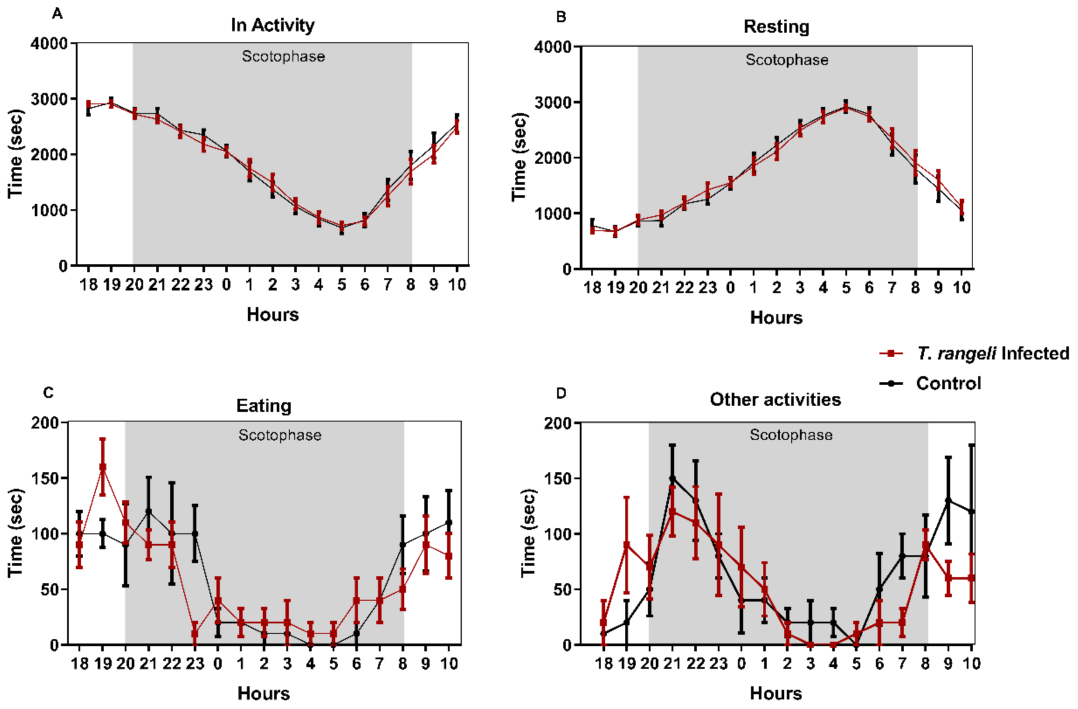

Supplementary Figure 4. Activity of mice infected or not with *Trypanosoma rangeli*. **A**. Time spent moving; **B**. Time spent resting; **C**. Time spent in contact with food; **D**. Time spent performing other activities. Data are presented as mean ± standard error of the mean (SEM) from 10 independent assays (GEE, not significant).
