# Supplementary material for: Trypanosoma rangeli turns infected mice more attractive to triatomine bugs
Source: Proc Biol Sci. 2026 Nov 11;293(2082):20262486. doi: 10.1098/rspb.2026.2486 (PMC13751123; doi:10.1098/rspb.2026.2486)
Supplement: Supplementary file 1 [file rspb.2026.2486.zip › rspb.2026.2486/rspb20262486_si_002.pdf]

Authors: Luan Felipe Santos; Marcelo Gustavo Lorenzo; Renan Pedra de Souza; Alessandra Aparecida Guarneri[1]
Title: *Trypanosoma rangeli* turns infected mice more attractive to triatomine bugs
Journal name: Proceedings B
Article DOI: 10.1098/rspb.2026.2486.

Supplementary statistical analysis

Binomial generalized linear mixed-effects models for host preference

Host preference was analyzed separately for the *Trypanosoma rangeli* and *Trypanosoma cruzi* experiments using binomial generalized linear mixed-effects models with a logit link. For each observation interval, the response was specified as the paired counts of insects found near the infected and control mice. Therefore, the model estimated the probability that an insect approaching either host was found near the infected mouse rather than the control mouse. Observation intervals in which no insects approached either mouse were excluded because host preference was undefined for those observations.
Time was expressed in hours and centered at its mean before model fitting. Consequently, the model intercept represented the log-odds of approaching the infected mouse at the average observation time, with a value of zero corresponding to equal preference for infected and control mice. The coefficient for centered time represented the change in log-odds of preference for the infected mouse for each additional hour of observation. Assay was included as a random intercept to account for repeated observations obtained from the same experimental unit.

For the *T. rangeli* experiments, the model included 170 observations from 10 independent assays. Model fit statistics were AIC = 275.8, BIC = 285.2, and log-likelihood = −134.9. The estimated variance of the assay-level random intercept was very small (variance = 1.58×10^{-9}; SD = 3.97×10^{-5}), indicating little residual between-assay heterogeneity after accounting for the fixed effects. The intercept was positive and statistically significant (β = 0.520, SE = 0.124, z = 4.203, $p < 0.001$), corresponding to odds of 1.68 and an estimated probability of 62.7% of approaching the infected mouse at the average observation time. The effect of time was not significant (β = 0.0175, SE = 0.0298, z = 0.587, p = 0.557; OR = 1.018 per hour), indicating no evidence that the relative preference changed over time.
For the *T. cruzi* experiments, the model included 411 observations from 15 independent assays. Model fit statistics were AIC = 891.4, BIC = 903.4, and log-likelihood = −442.7. The assay-level random-intercept variance was again negligible (variance = 3.67×10^{-10}; SD = 1.92×10^{-5}). The intercept was slightly negative (β = −0.103, SE = 0.052, z = −2.001, p = 0.045), corresponding to odds of 0.90 and an estimated probability of 47.4% of approaching the infected mouse at the average observation time, or conversely 52.6% for the control mouse. The effect of time was essentially null (β = 0.00036, SE = 0.0134, z = 0.027, p = 0.978; OR = 1.000 per hour), providing no evidence that preference changed during the observation period.

Computationally, all analyses were performed in R using the glmmTMB package. Models were fitted by maximum likelihood with Laplace approximation for integration over the random effects. Before model fitting, the data were reshaped so that each row represented one assay at one observation interval, preserving the paired infected and control counts within the same experimental unit. Time was converted to hours and mean-centered within each dataset prior to analysis. Model adequacy was assessed using simulation-based residual diagnostics implemented in the DHARMa package, including tests of residual uniformity, dispersion, and outliers. Model-estimated probabilities and corresponding 95% confidence intervals were obtained using the ggeffects package and transformed to the response scale for graphical presentation. The same modelling and diagnostic workflow was applied independently to the *T. rangeli* and *T. cruzi* datasets. Statistical significance was assessed using two-sided tests with $p < 0.05$.

Supplementary Table S1. Binomial mixed-effects models of host preference

**Parameter**	***T. rangeli***	***T. cruzi***
Independent assays, n	10	15
Observations, n	170	411
AIC	275.8	891.4
BIC	285.2	903.4
Log-likelihood	−134.9	−442.7
Residual df	167	408
Random-intercept variance	1.58×10^{-9}	3.67×10^{-10}
Random-intercept SD	3.97×10^{-5}	1.92×10^{-5}
Intercept, β (SE)	0.520 (0.124)	−0.103 (0.052)
z	4.203	−2.001
p	<0.001	0.045
OR	1.682	0.902
Estimated P(infected) at mean time	62.7%	47.4%
Time, β (SE)	0.0175 (0.0298)	0.00036 (0.0134)
z	0.587	0.027
p	0.557	0.978
OR per additional hour	1.018	1.000

Table note: The intercept corresponds to host preference at the mean observation time because time was mean-centered. Probabilities above 50% indicate preference for the infected mouse, whereas probabilities below 50% indicate preference for the control mouse. The time coefficient represents the change in the log-odds of approaching the infected mouse per additional hour.

Generalized Linear Mixed Model (GLMM, Poisson distribution) for insect approach behavior in the predation rate experiment.

Four alternative modelling strategies were evaluated to account for the progressive reduction in insect availability caused by predation over the course of each trial: (i) no adjustment for predation; (ii) inclusion of total preyed insects as a covariate; (iii) an offset based on the mean insect availability during the trial; and (iv) an offset based on the final insect availability at the end of the trial. Model (iii) was selected as the primary model reported in the main text, as it most realistically captures the dynamics of insect availability throughout the trial while keeping the group effect significant. Models (i), (ii), and (iv) are reported below for comparison and transparency.

Supplementary Table S2. Model (i): No adjustment for predation

glmer(Count ~ Group + (1|Trial) + (1|Time), family = poisson, data = data)

**Predictors**	**Rate Ratio**	**95% CI**	**p**
Intercept	2.54	1.55 – 4.17	<0.001
Group [Control] (ref: Infected)	0.83	0.73 – 0.94	0.004
**Random Effects**			
σ^2 (residual)	0.25		
τ00 Time	0.70		
τ00 Trial	0.17		
ICC	0.78		

N Trial = 9; N Time = 17; Observations = 306; Marginal R^2 / Conditional R^2 = 0.008 / 0.783.

Supplementary Table S3. Model (ii): Total preyed insects included as covariate

glmer(Count ~ Group + Total_preyed + (1|Trial) + (1|Time), family = poisson, data = data)

**Predictors**	**Rate Ratio**	**95% CI**	**p**
Intercept	2.31	1.35 – 3.95	0.002
Group [Control] (ref: Infected)	0.79	0.66 – 0.94	0.007
Total preyed insects	1.01	0.98 – 1.04	0.368
**Random Effects**			
σ^2 (residual)	0.25		

τ00 Time	0.70		
τ00 Trial	0.17		
ICC	0.78		

N Trial = 9; N Time = 17; Observations = 306; Marginal R^2 / Conditional R^2 = 0.012 / 0.783.

Supplementary Table S4. Model (iii): Offset = log(mean insect availability) — model reported in main text

glmer(Count ~ Group + (1|Trial) + (1|Time), offset = log(Mean_availability), family = poisson, data = data)

**Predictors**	**Rate Ratio**	**95% CI**	**p**
Intercept	0.05	0.03 – 0.09	<0.001
Group [Control] (ref: Infected)	0.87	0.77 – 0.99	0.034
**Random Effects**			
σ^2 (residual)	2.61		
τ00 Time	0.70		
τ00 Trial	0.18		
ICC	0.25		

N Trial = 9; N Time = 17; Observations = 306; Marginal R^2 / Conditional R^2 = 0.001 / 0.254. Mean availability = (Initial available + Final available) / 2, where Final available = 50 − Number preyed.

Supplementary Table S5. Model (iv): Offset = log(final insect availability)

glmer(Count ~ Group + (1|Trial) + (1|Time), offset = log(Final_availability), family = poisson, data = data)

**Predictors**	**Rate Ratio**	**95% CI**	**p**
Intercept	0.06	0.04 – 0.10	<0.001
Group [Control] (ref: Infected)	0.92	0.81 – 1.05	0.231
**Random Effects**			
σ^2 (residual)	2.49		

τ00 Time	0.70		
τ00 Trial	0.19		
ICC	0.26		

N Trial = 9; N Time = 17; Observations = 306; Marginal R^2 / Conditional R^2 = 0.000 / 0.265. Final available = 50 − Number preyed.

Summary

Across all four modelling strategies, the direction and statistical significance of the group effect remained largely consistent, except when adjusting for final insect availability (Model iv), which diluted the group effect due to over-penalizing trials with higher early predation. The mean-availability offset (Model iii) was therefore retained as the primary model, as it best reflects the realistic dynamics of insect availability throughout each trial.

Generalized Estimating Equations (GEE) model for mouse nocturnal activity.

Gaussian distribution, identity link function, exchangeable correlation structure. The response variable is time (in seconds) spent in each behavioral category. Group (infected vs. control) and behavioral category (playing [reference], eating, resting, active time) were included as fixed effects, along with their interaction. An intermediate model that additionally tested the effect of Hour was evaluated and found non-significant for all hour levels; Hour was therefore not retained in the final model reported below.

Supplementary Table S6. Final model

*geeglm(Time ~ Group * Activity, id = ID, family = gaussian, corstr = "exchangeable", data = data)*

**Predictors**	**Estimate**	**95% CI**	**p**
Intercept	59.19	19.80 – 98.58	0.003
Group [Infected] (ref: Control)	-4.85	-75.19 – 65.49	0.892
Activity [Eating] (ref: Playing)	1.76	-17.58 – 21.11	0.858
Activity [Resting] (ref: Playing)	1646.47	1547.05 – 1745.89	<0.001
Activity [Active time] (ref: Playing)	1836.18	1735.47 – 1936.88	<0.001

N ID = 6; Observations = 816. Estimates represent mean differences in time (seconds) relative to the reference category (Playing). Group × Activity interaction terms were tested and removed from the final model due to non-significance (all $p > 0.05$).
